# Re-employment, job quality, health and allostatic load biomarkers: prospective evidence from the UK Household Longitudinal Study

**DOI:** 10.1093/ije/dyx150

**Published:** 2017-08-10

**Authors:** Tarani Chandola, Nan Zhang

**Affiliations:** Cathie Marsh Institute and Social Statistics, School of Social Science, University of Manchester, Oxford Road, Manchester M13 9PL, UK

**Keywords:** Re-employment, unemployment, biomarkers, well-being, job quality, allostatic load

## Abstract

**Background:**

There is little evidence on whether becoming re-employed in poor quality work is better for health and well-being than remaining unemployed. We examined associations of job transition with health and chronic stress-related biomarkers among a population-representative cohort of unemployed British adults.

**Methods:**

A prospective cohort of 1116 eligible participants aged 35 to 75 years, who were unemployed at wave 1 (2009/10) of the UK Household Longitudinal Study, were followed up at waves 2 (2010/11) and 3 (2011/12) for allostatic load biomarkers and self-reported health. Negative binomial and multiple regression models estimated the association between job adversity and these outcomes.

**Results:**

Compared with adults who remained unemployed, formerly unemployed adults who transitioned into poor quality jobs had higher levels of overall allostatic load (0.51, 0.32–0.71), log HbA1c (0.06, <0.001–0.12), log triglycerides (0.39, 0.22–0.56), log C-reactive protein (0.45, 0.16–0.75), log fibrinogen (0.09, 0.01–0.17) and total cholesterol to high-density lipoprotein (HDL) ratio (1.38, 0.88–1.88). Moreover, physically healthier respondents at wave 1 were more likely to transition into good quality and poor quality jobs after 1 year than those who remained unemployed.

**Conclusions:**

Formerly unemployed adults who transitioned into poor quality work had greater adverse levels of biomarkers compared with their peers who remained unemployed. The selection of healthier unemployed adults into these poor quality or stressful jobs was unlikely to explain their elevated levels of chronic stress-related biomarkers. Job quality cannot be disregarded from the employment success of the unemployed, and may have important implications for their health and well-being.

## Introduction

Unemployment is associated with poor health^1–7^ and appropriate work can bring health and well-being benefits.^8^ There are health benefits of transitioning from unemployment into employment/re-employment.^6^^,^^9–15^ There is also some evidence that job quality is important for health and well-being, although other studies suggest that people in poor quality jobs are still better off in terms of life satisfaction and well-being than those who remain unemployed.^10^^,^^16^ However, poor quality jobs which combine several psychosocial stressors could be as bad for health as being unemployed,^17^^,^^18^ and transitions from unemployment to poor quality jobs may be even more detrimental to health than remaining unemployed.^19^ Thus the quality of job, including the presence of stressors such as insecurity, low autonomy and poor job satisfaction, may be important in determining whether transitioning into work benefits or harms health.

The existing evidence on re-employment and health/well-being relies on self-reported measures for both concepts, both of which may be biased by mood or personality traits. Biomarkers measuring the physiological consequences of chronic stress like allostatic load^20^ can provide insights into a person’s health and well-being which are different from self-reported measures. Unemployment is associated with adverse levels of common biomarkers measured in longitudinal studies.^21–23^ Additionally, most studies on job quality and health measure job satisfaction, although there are additional dimensions such as job security and work autonomy.^24^ However, despite the methodological weakness of existing studies on re-employment and health, government policy on employment and health often assumes that the benefits of work outweigh any ‘risks’of work and the adverse effects of unemployment.^8^^,^^25^

The relationship between health and employment may be bidirectional:^11^ unemployment may cause poor health, and poor health may increase the probability of unemployment. Observational studies of work and health need to take account of the selection process into employment. Health-related selection factors are particularly important, due to the healthy worker effect–adults with poor health are selected out of the job market and their health may be a barrier to employment/re-employment.^15^^,^^26–28^

The aim of the study was to examine the association of job transition with health and chronic stress-related biomarkers among a cohort of unemployed British adults. We were particularly interested in comparing the health of those who remained unemployed with those who transitioned to poor quality work, and examining whether there was positive (or negative) health selection into good (or poor) quality jobs.

## Methods

This study draws upon data from the first three waves of the *Understanding Society*, the UK Household Longitudinal Study (UKHLS): a nationally representative longitudinal study that began in 2009, recruiting over 100 000 individuals in 40 000 households.^29^ Further details of the study are available elsewhere.^30^ In 2010–12 (waves 2 and 3), adult respondents were invited to take part in a nurse health assessment interview which collected a range of physiological measures and blood samples.^31^ A representative subsample of 15 591 adults took part in a nurse health assessment, with a response rate of 58.6%.^31^ Among those participants, 10 175 (response rate 38.2%) gave a blood sample and had data on at least one biomarker.^32^

### Sample

**Figure 1:**
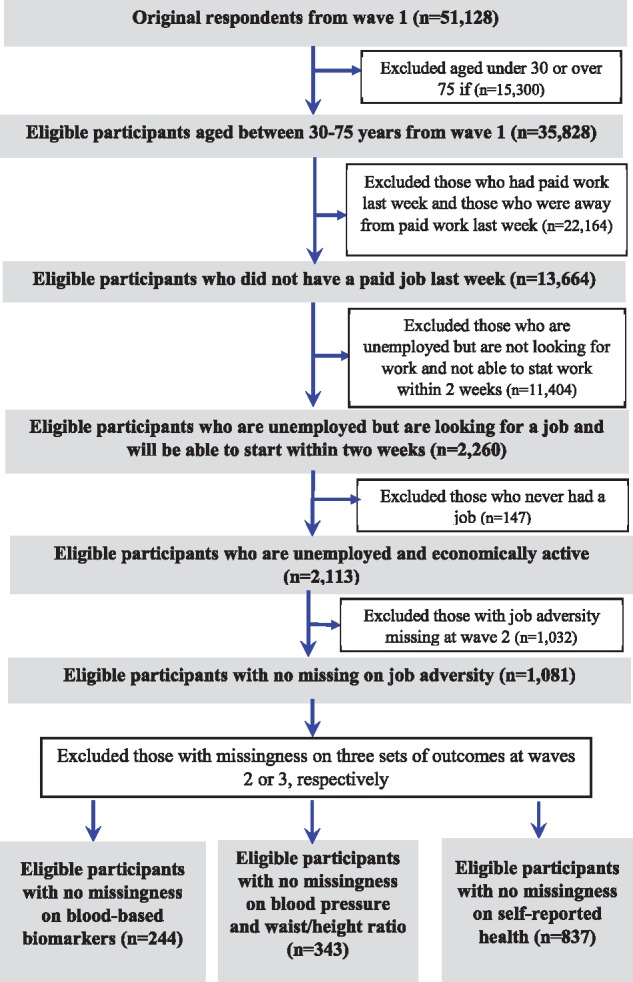
Flow chart showing the selection of UKHLS participants for the analyses.

The selection of participants for this study is summarized in Figure 1. Of the 51 128 original participants at wave 1, there were 35 828 aged between 30 and 75 years. Participants were excluded if they were in paid work or if they were away from a paid job in the previous week (*n* = 22 164), if they were not looking for work and were not be able to start work within 2 weeks (*n* = 11 404), if they never had a job or were economically inactive (*n* = 147) or if they had missing data on job quality measures at wave 2 (*n* = 1032). Among eligible participants at wave 1 (*n* = 1081), we further excluded those with missing data on outcome variables at wave 2. Thus, the final analytical samples comprised 244 adults for blood-based biomarkers as outcomes, 343 adults for other biomarkers such as blood pressure and anthropometry and 837 adults for self-reported health. 

### Outcomes

We used the concept of allostatic load to measure chronic stress-related biomarkers.^33^ Detailed information on each biomarker and its application can be found in Table 1.

**Table 1:** Biomarkers from Understanding Society used in this study^32^

Biomarkers used in this study	Units	Computation and transformation	Application
HbA1c	Glycosylated haemoglobin (HbA1c)(mmol/mol)	Log-transformation of HbA1c	Undiagnosed or poorly managed diabetes
Triglycerides	Triglycerides (mmol/l)	Log-transformation of triglycerides	‘Fat in the blood’ associated with heart disease
C-creative protein	C-creative protein (CRP) (mg/l)	Log-transformation of CRP	Measures of inflammation—due to injury or infection—acute or chronic—response to stress
Fibrinogen	Clauss fibrinogen (g/l)	Log-transformation of fibrinogen	
DHEA-S	Dehydroepiandrosteronesulphate (DHEA-S) (µmol/l)	Log-transformation of DHEA-S	Associated cardiovascular disease, muscle strength, cognition
Creatinine clearance rate	Creatinine(µmol/l)	[140-age (years)] x weight (kg) x f/serum creatinine (µmol/l) where f = 1.23 for males and 1.04 for females^42^	Kidney diseases: increases with age, associated other diseases
Insulin-like growth factor 1	Insulin-like growth factor 1(IGF-1)(nmol/l)	Log-transformation of IGF-1(nmol/l)	Growth and development—associated diet, diabetes and cancer
Total cholesterol-to- HDL ratio	Total cholesterol (mmol/l) HDL cholesterol (mmol/l)	Total cholesterol (mmol/l)/HDL cholesterol (mmol/l)	‘Fat in the blood’ associated with heart disease
Systolic blood pressure	Systolic blood pressure(mmHg)	Log-transformation of (systolic blood pressure + n) where n = 10 if on blood pressure medication and 0 if not on blood pressure medication^43^	Hypertension and associated with cardiovascular disease
Diastolic blood pressure	Diastolic blood pressure(mmHg)	Log-transformation of (diastolic blood pressure + n) where n = 5 if on blood pressure medication and 0 if not on blood pressure medication^43^	Hypertension and associated with cardiovascular disease
Waist-to-height ratio	Waist (cm) Height (cm)	Waist (cm)/height (cm)	Body fat distribution and a predictor of metabolic consequences independent of overall adiposity
Pulse	Pulse (beats per minute)	Log-transformation of pulse	Heart rate
Allostatic load	Number of biomarker risk factors (range 0–12)	Sum of the above 12 biomarkers where the risk quartile was coded as 1 and the remaining quartiles were coded as 0	Associated with cardiovascular, metabolic and immune system diseases

The allostatic load index has previously been used to measure health-related effects of work stress.^20^ This index was originally based on data from 10 physiological or physical measurements across the cardiovascular, metabolic and immune systems.^34^ We used 12 biomarkers measured in the UKHLS [insulin growth factor 1, creatinine clearance rate and dehydroepiandrosterone sulphate (DHEA-S) with lower values indicating higher risk; Clauss fibrinogen, C-reactive protein, ratio of total to HDL cholesterol, triglycerides, HbA1c, pulse, systolic and diastolic blood pressures, and waist-to-height ratio; with higher values indicating higher risk] to construct the index. Highest (sex-specific) quartiles of Clauss fibrinogen, C-creative protein, ratio of total cholesterol to HDL cholesterol, triglycerides, HbA1c, systolic and diastolic blood pressures, and waist-to-height ratio were coded as 1 and the remaining quartiles as 0. The lowest quartiles of insulin growth factor 1 (IGF-1), creatinine clearance rate and DHEA-S were coded as 1 and the remaining as 0. The allostatic load index was a summary of the 12 grouped biomarkers. Eight of the 12 biomarkers were blood based, and four non-blood based biomarkers were additionally collected during the nurse health assessment: pulse, blood pressures and waist-to-height ratio. Pulse and systolic and diastolic blood pressures were measured using the Omoron Hem 907 electronic sphygmomanometer. In addition, we used waist-to-height ratio (WHR) as a replacement for waist-to-hip ratio to measure body fat distribution.^35^^,^^36^ Log-transformation was used to reduce skewness for the non-normally distributed biomarkers.

Two self-reported health outcomes were examined, the Short-Form Physical and Mental Health Composite Scale scores (SF-12 PCS and SF-12 MCS) which measure physical and mental health functioning, respectively, with scores ranging from 0 (low functioning) to 100 (high functioning). In addition to the health and biomarkers as dependent variables, we analysed the association of job transition with monthly total household net income (log transformed). 

### Job quality

Levels of job quality were derived based on three dimensions of job quality-earnings quality, labour market security and quality of the working environment.^24^

- Job satisfaction was measured on a 7-point Likert-scale (where 1 = completely dissatisfied and 7 = completely satisfied), with 4 as the cut-off to define low job satisfaction.
- Job anxiety was derived as the mean of six questions on job-related well-being, e.g. how much of the time in the past week one felt tense/uneasy/worried/depressed/gloomy/miserable about a job. Each question was scored 1 (never), 2 (occasionally), 3 (some of the time), 4 (most of the time) and 5 (all of the time). A cut-off of 2 or higher reflects some job anxiety.
- Job autonomy was derived from the mean of five questions on how much influence a participant has over tasks, workplace, work manner, task order and working hours in his/her current job. Each question was measured on a 4-point scale from 1 (a lot), 2 (some), 3 (a little) to 4 (none). A cut-off of 2 or higher represents a low-autonomy job.
- Job insecurity was measured by a question on ‘how likely you think it is that you will lose your job during the next 12 months’. The four response categories were grouped into: low job security (very likely or likely) and high job security (unlikely or very unlikely).
- Low job pay was defined as being in the lowest quartile of hourly pay, which was calculated from a person’s usual gross pay per month and number of hours per week.

An overall job quality variable was created by cross-classifying job transition with the five job quality variables. We derived four groups: remained unemployed; employed in a good quality job (with no adverse job quality measure); employed in a job with only one adverse job quality measure; and employed with at least two adverse job quality measures.

### Covariates

We included baseline (wave 1) sociodemographic, socioeconomic characteristics and health as covariates in the regression analyses. These included age (categorized as: 30–39 years; 40–49 years; and 50–75 years), gender (male and female), ethnicity (White British and non-White), number of children within the household, household size, log household net income, year since last employed (before the start of the ‘Great Recession’ in 2008, 2008 and 2009–10), highest academic qualification (degree level or higher, A level and GCSE qualifications, and other and no qualification), housing tenure (owned house, council rented house and private rented), marital status (married, single, other, separated and divorced or widowed), body mass index (BMI), self-reported cardiovascular disease (CVD) or diabetes, SF-12 PCS/SF-12 MCS (at wave 1), self-reported long-term illness or impairment, General Health Questionnaire (GHQ-12) score with a cut-off of 4 and higher reflecting minor psychiatric morbidity, and the number of prescribed medicines taken (categorized as 0, 1–2, and 3 medications or more).

### Analysis

As allostatic load is a count of biomarker risk indicators, negative binomial regression models were used to estimate the association with job quality after controlling for baseline covariates. Multiple linear regression models were used for the other dependent variables (the log transformed biomarkers, SF-12 PCS/MCS and log household net income) Multinomial logistic regression was used to examine which risk factors at wave 1 predicted job adversity at wave 2. Three different weights were employed, depending on the dependent variables to adjust for unequal selection probabilities and cross-sectional and longitudinal non-response.^31^ Longitudinal blood sample weights were used for the blood-based biomarkers and allostatic load. Longitudinal nurse visit weights were used for pulse, blood pressure and waist-height ratio. Longitudinal adult interview weights were used for two self-reported health outcomes and household net income. All statistics were calculated using the *svy* commands in Stata version 13 (37) which takes account of sample selection, non-response bias and the complex survey design for point estimates and variance estimation. Missing outcome data, particularly the biomarker data at wave 2 which were only collected for a UKHLS subsample, were replaced with the equivalent data at wave 3 where available.

## Results

Table 2 displays the distribution of all the covariates and health outcomes by job adversity (at wave 2) for the cohort members (who were all unemployed at wave 1). The weighted mean (for continuous covariates) and percentages (for categorical covariates) by levels of job adversity are shown. Older adults (aged 50–75) were most likely to remain unemployed (51% of those who remained unemployed were aged 50–75) and least likely to transition into a good quality job (only 6% of those who transitioned into good quality jobs were aged 50–75). Women, adults with degree or higher qualifications and those living in their own homes were least likely to remain unemployed. Adults with baseline health conditions (CVD/diabetes, more prescribed medications, or long-term illness/impairment), higher BMI or lower SF-12 physical or mental health, and who were last employed before 2008, were most likely to remain unemployed.

**Table 2:** Distribution of covariates, health outcomes and biomarkers by job adversity among participants aged 30–75 years from Understanding Society, UK

	Remained unemployed		Good quality job		One adverse measure		At least two adverse measures	
Variables	*n*	Weighted %/mean	*n*	Weighted %/mean	*n*	Weighted %/mean	*n*	Weighted %/mean
Covariates								
Age range, %								
30–39 years	192	22.8	28	48.3	45	33.6	48	38.4
40–49 years	213	26.4	25	46.2	49	40.8	51	20.5
50–75 years	329	50.8	16	5.5	41	25.6	44	41.2
Sex, %								
Male	415	53.8	34	38.6	70	52.8	81	47.6
Female	319	46.2	35	61.4	65	47.2	62	52.4
Highest qualification, %								
Degree + higher	171	26.4	37	59.3	40	38.5	43	32.7
A level + GCSE	315	36.3	27	40.8	63	48.6	55	35.9
Other + no qualification	248	37.3	5	0	32	12.9	45	31.4
Housing tenure, %								
Owned	345	42.4	44	67.8	78	49.7	63	54.3
Council house	264	38.0	13	27.1	35	31.7	48	26.8
Rented	123	19.6	12	5.1	22	18.6	31	18.9
Marital status, %								
Married	327	36.8	30	36.0	70	35.0	71	38.6
Single	237	40.5	25	55.9	35	42.0	43	34.0
Separated, divorced or widowed	170	22.7	14	8.2	30	23.0	29	27.3
BMI, mean (SD)	302	28.0 (6.0)	26	25.9 (4.0)	50	26.3 (3.9)	47	27.9 (4.6)
Has CVD and/or diabetes, %								
No	525	67.5	51	75.2	102	81.4	121	87.8
Yes	209	32.5	18	24.8	33	18.6	21	12.2
SF-12 physical component score, mean (sd)	730	49.3 (11.3)	69	49.3 (9.2)	135	52.8 (8.5)	143	50.1 (7.5)
SF-12 mental component score, mean (sd)	730	46.1 (11.8)	69	45.6 (12.6)	135	49.1 (11.1)	143	48.6 (10.9)
Long-term illness/impairment, %								
No	394	52.3	45	51.6	102	76.5	98	74.2
Yes	340	47.7	24	48.4	33	23.5	45	25.8
General Health Questionnaire (GHQ-12) score, %								
Non-distressed (0 ≤ GHQ-12) score ≤ 3)	462	66.7	45	61.2	88	73.9	79	65.4
Distressed (GHQ-12 score ≥ 4)	189	33.3	19	38.8	31	26.1	47	34.6
Number of prescribed medicines taken								
0	115	39.3	20	59.5	25	51.9	24	53.4
1–2 medicines	98	29.0	7	22.2	16	29.1	12	25.5
≥3 medicines	104	31.7	4	18.3	10	19.0	14	21.1
Race/ethnicity, %								
White British	176	13.7	22	10.9	36	16.2	47	20.4
Non-White	557	86.3	47	89.1	99	83.8	96	79.6
Log total household net income mean (SD)	734	7.1 (1.0)	69	7.5 (0.6)	135	7.3 (1.3)	143	6.3 (2.3)
Year of last employment, %								
Before 2008	392	58.6	14	17.7	30	31.8	25	15.4
2008	136	21.0	17	16.3	28	16.6	40	28.1
2009–10	185	20.4	36	66.0	76	51.6	76	56.5
Number of children in household, mean (SD)	734	0.6 (1.1)	69	0.8 (1.1)	135	0.8 (1.0)	143	0.5 (1.1)
Number of people in household, mean(SD)	734	2.5 (1.4)	69	2.8 (1.2)	135	3.0 (1.3)	143	2.4 (1.3)
Biomarkers (wave 2) as outcomes, mean (SD)								
Allostatic load^b^	204	2.9 (2.0)	20	1.5 (1.9)	33	2.1 (1.7)	26	3.6 (2.5)
Log HbA1c^b^	195	3.6 (0.2)	19	3.6 (0.2)	33	3.6 (0.1)	25	3.7 (0.3)
Log triglycerides^b^	206	0.5 (0.6)	20	0.3 (0.4)	32	0.5 (0.6)	26	0.7 (0.5)
Log C-creative protein^b^	196	0.6 (0.9)	18	0.02 (0.8)	31	0.5 (1.1)	22	0.9 (0.8)
Log fibrinogen^b^	206	1.0 (0.2)	20	0.9 (0.3)	33	0.9 (0.3)	27	1.1 (0.2)
Log DHEA-S^b^	206	1.3 (0.8)	20	1.6 (0.6)	32	1.6 (0.6)	26	1.3 (0.6)
Creatinine clearance rate^b^	202	120.6 (42.9)	20	132.9 (25.4)	32	116.7 (28.2)	26	109.5 (32.1)
Log insulin-like growth factor 1^b^	201	2.8 (0.3)	20	2.9 (0.3)	32	2.9 (0.2)	26	2.7 (0.3)
Total cholesterol-to-HDL ratio^b^	206	4.1 (1.7)	20	3.4 (1.0)	32	4.4 (1.5)	26	4.8 (2.1)
Log systolic blood pressure	244	4.83 (0.13)	23	4.78 (0.10)	38	4.79 (0.11)	42	4.85 (0.12)
Log diastolic blood pressure	244	4.32 (0.14)	23	4.31 (0.14)	38	4.31 (0.13)	42	4.34 (0.13)
Waist-to-height ratio	311	0.58 (0.09)	30	0.55 (0.08)	51	0.56 (0.08)	50	0.59 (0.08)
Log pulse	244	4.25 (0.16)	23	4.26 (0.13)	38	4.27 (0.16)	42	4.23 (0.16)
Self-reported health (wave 2) as outcomes, mean(SD)								
SF-12 physical component score^a^	572	48.9 (11.0)	58	52.1 (10.0)	102	51.3 (9.5)	105	51.5 (10.1)
SF-12 mental component score^a^	572	46.2 (11.9)	58	53.2 (7.4)	102	51.2 (7.4)	105	48.4 (11.0)

Figures are means for continuous variables and percentages for categorical variables that are weighted with sampling weights. Sample sizes are not weighted.

SD, standard deviation; GCSE, General Certification of Secondary Education; CVD, cardiovascular disease.

^a^Used longitudinal main adult interview weights at wave 3.

^b^Used combined longtidunal blood interview weight at wave 2 and wave 3; the rest of health outcomes used longitudinal nurse visit weights at wave 3.

Looking at the allostatic load biomarkers, there was a clear pattern of the highest levels for adults who transitioned into poor quality work, with the exception of the measures where higher levels indicated better functioning (creatinine clearance rate, IGF-1 and DHEA-S). Adults who transitioned into good quality jobs had the lowest levels of biomarkers, with the exception of the creatinine clearance rate, IGF-1 and DHEA-S, where their levels were the highest. Respondents who remained unemployed tended to have the poorest (lowest) SF-12 physical and mental scores of at wave 3.

**Figure 2:**
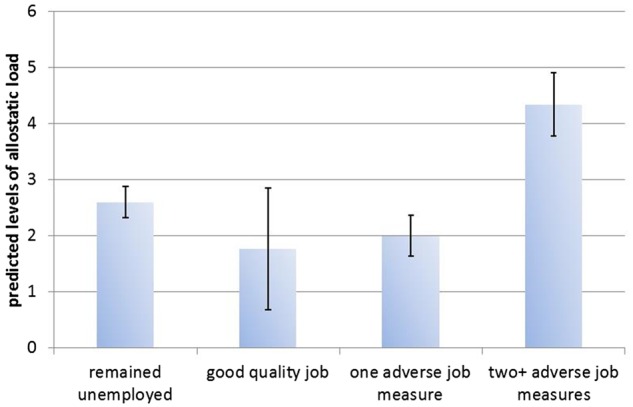
Predicted levels of allostatic load by job transition and adversity, estimated from coefficients in Table 3 and Supplementary Table 1a.

Table 3 reports the results of the regression models predicting the wave 3 allostatic load biomarkers and SF-12 physical and mental health scores, with job adversity as the main explanatory variable. The full models with all the covariates are shown in Supplementary Tables 1–3, available as Supplementary data at *IJE* online. Compared with cohort members who remained unemployed at wave 2, those who transitioned into poor quality work (with at least two adverse job quality measures) had higher levels of overall allostatic load (0.51, 0.32–0.71), log HbA1c (0.06, <0.001–0.12), log triglycerides (0.39, 0.22–0.56), log CRP (0.45, 0.16–0.75), log fibrinogen (0.09, 0.01–0.17) and total cholesterol to HDL ratio (1.38, 0.88–1.88). The creatinine clearance rate was lowest among those in poor quality work. Respondents who transitioned into good quality work tended to have lower levels of allostatic load biomarkers, with the exception of those biomarkers where higher levels indicate better functioning: DHEA-S and the creatinine clearance rate. The predicted levels of allostatic load (from Table 3) by job transition are shown in Figure 2; those who transitioned into poor quality work had levels of allostatic load that were over 1.5 times higher compared with those who remained unemployed. We also examined the association between allostatic load and each job quality dimension (Supplementary Table 4, available as Supplementary data at *IJE* online). Respondents who transitioned into poor quality work, as measured by low pay, low job satisfaction, low job control and high job anxiety, had higher levels of allostatic load compared with their peers who remained unemployed.

**Table 3:** Associations between job adversity, health outcomes, allostatic load biomarkers and household income among participants aged 30–75 years from Understanding Society, UK. Negative binomial regression coefficients (and 95% CI) of allostatic load and multiple regression coefficients (and 95% CI) of allostatic load biomarkers, self-reported health and household income, regressed on job adversity and adjusted for covariates

Outcomes	Job adversity(reference: remained unemployed)						
	Good quality job		One adverse measure		At least two adverse measures		Overall *P*-value
	β	95% CI	β	95% CI	β	95% CI	
Biomarkers							
Allostatic load	−0.387	(−1.003, 0.230)	−0.262	(−0.476, −0.047)	0.512	(0.320, 0.706)	<0.001
Log HbA1c	0.004	(−0.109, 0.117)	−0.020	(−0.081, 0.042)	0.057	(−0.004, 0.117)	0.104
Log triglycerides	−0.165	(−0.435, 0.105)	0.029	(−0.143, 0.201)	0.389	(0.220, 0.558)	<0.001
Log C-creative protein	−0.353	(−0.852, 0.146)	0.183	(−0.161, 0.527)	0.454	(0.158, 0.749)	0.0008
Log fibrinogen	−0.081	(−0.191, 0.028)	−0.143	(−0.237, −0.049)	0.089	(0.007, 0.170)	<0.001
Log DHEA-S	0.182	(−0.149, 0.513)	0.012	(−0.233, 0.257)	−0.082	(−0.305, 0.142)	0.360
Creatinine clearance rate	−2.634	(−19.631, 14.362)	−1.645	(−13.410, 10.120)	−25.968	(−35.910, −16.026)	<0.001
Total cholesterol-to-HDL ratio	−0.151	(−0.852, 0.551)	−0.025	(−0.468, 0.518)	1.377	(0.876, 1.878)	<0.001
Log insulin-like growth factor 1	0.025	(−0.205, 0.256)	0.067	(−0.041, 0.174)	0.016	(−0.088, 0.120)	0.670
Log systolic blood pressure	−0.023	(−0.068, 0.023)	−0.019	(−0.052, 0.014)	0.028	(−0.018, 0.074)	0.200
Log diastolic blood pressure	−0.029	(−0.086, 0.027)	−0.010	(−0.056, 0.037)	0.010	(−0.071, 0.091)	0.688
Waist-to-height ratio	−0.020	(−0.041, 0.001)	−0.007	(−0.022, 0.008)	−0.003	(−0.022, 0.017)	0.253
Log pulse	−0.010	(−0.120, 0.099)	−0.010	(−0.075, 0.056)	−0.029	(−0.107, 0.04)	0.911
Self-reported health							
SF-12 physical component score	−0.701	(−4.611, 3.209)	−0.490	(−1.671, 0.691)	1.914	(−3.599, 7.426)	0.784
SF-12 mental component score	5.541	(−2.841, 13.923)	3.103	(0.966, 5.240)	2.299	(−2.406, 7.005)	0.035
Log total household net income	0.402	(−0.146, 0.949)	0.320	(−0.008, 0.648)	0.408	(0.037, 0.779)	0.201

Fully-adjusted models were fitted by adjusting for age, gender, highest qualification, housing tenure, marital status, BMI, has CVD and/or diabetes or not, SF-12 physical health composite scale scores, SF-12 mental health composite scale scores, long-term illness or impairment, General Health Questionnaire (GHQ-12) score, number of prescribed medicines taken, log transformation of household net income, race/ethnicity, number of children within household, number of people within household and year of latest employment.

Looking at the SF-12 physical and mental health scores as the outcomes, transitioning into any type of job was not associated with an improvement in physical health. Good quality work was associated with an improvement in mental health scores compared with remaining unemployed, but there were no differences in SF-12 mental health scores between those who transitioned into poor quality work and those who remained unemployed. Remaining unemployed was associated with lower total net household income compared with those who transitioned into any job.

Table 4 reports the results of the multinomial logit models predicting job adversity levels at wave 2 from the wave 1 covariates. Older adults (aged 50–75) were less likely to transition into any job (regardless of job adversity) and more likely to remain unemployed when compared with those who transitioned into any job. Gender differences in transitioning into job quality were small. Respondents with other/no qualifications were least likely to transition into good quality jobs. Similarly, those living in council rented homes were least likely to transition into good quality jobs. Marital status did not predict job quality transitions, and neither did BMI, having CVD/diabetes, GHQ distress, ethnicity, household net income, number of children in the household or household size. Those with better physical health (higher SF-12 PCS) were more likely to transition into either good or poor quality jobs, whereas those with a long-standing illness/impairment were unlikely to transition into any job and most likely to remain unemployed when compared with those without any illness/impairments. Those who had been unemployed the longest (since before the ‘Great Recession’ in 2008) were also the most likely to remain unemployed.

**Table 4:** Multinomial logit models of job adversity at wave 2 regressed on covariates from Understanding Society, the UK. Figures are coefficients and 95% *CI.* Only bivariate associations are reported

Risk factors	Job adversity(reference: remained unemployed)						
	Good quality job		One adverse measure		At least two adverse measures		Overall *p*-value
	*β*	*95%CI*	*β*	*95%CI*	*β*	*95%CI*	
Age range (Ref: 30–39 years)							0.011
40–49 years	−0.317	(−1.041, 0.408)	−0.078	(−0.644, 0.490)	−0.271	(−0.881, 0.339)	
50–75 years	−1.185	(−1.958, −0.412)	−0.648	(−1.183, −0.112)	−0.731	(−1.323, −0.138)	
Sex (Ref: Male)							0.443
Female	0.368	(−0.210, 0.946)	0.274	(−0.203, 0.751)	0.047	(−0.511, 0.604)	
Highest qualification (Ref: Degree + Higher)							0.008
A level + GCSE	−0.971	(−1.622, −0.320)	−0.320	(−0.863, 0.223)	−0.297	(−0.909, 0.314)	
Other + No qualification	−2.241	(−3.484, −0.999)	−0.817	(−1.559, −0.075)	−0.335	(−1.011, 0.341)	
Housing tenure (Ref: Owned)							0.014
Council house	−1.131	(−1.786, −0.475)	−0.597	(−1.143, −0.050)	−0.189	(−0.838, 0.459)	
Rented	−0.555	(−1.375, 0.265)	−0.324	(−1.027, 0.380)	0.294	(−0.311, 0.899)	
Marital status (Ref: Married)							0.634
Single	−0.141	(−0.846, 0.564)	−0.469	(−0.986, −0.049)	−0.310	(−0.954, 0.333)	
Separated, divorced or widowed	−0.264	(−0.876, 0.347)	−0.161	(−0.797, 0.476)	−0.214	(−0.790, 0.361)	
BMI	−0.043	(−0.158, 0.072)	−0.044	(−0.118, 0.029)	0.044	(−0.056, 0.144)	0.300
Has CVD and/or diabetes (Ref: No)							0.110
Yes	−0.169	(−0.693, 0.355)	−0.166	(−0.783, 0.452)	−0.944	(−1.693, −0.195)	
SF-12 physical health composite scale scores	0.038	(0.005, 0.072)	0.025	(−0.007, 0.057)	0.028	(0.002, 0.054)	0.021
SF-12 mental health composite scale scores	0.020	(0.001, 0.039)	0.020	(−0.004, 0.045)	0.002	(−0.019, 0.024)	0.108
Has long-term illness or impairment (Ref: No)							0.004
Yes	−0.511	(−1.078, 0.056)	−0.829	(−1.404, −0.253)	−0.623	(−1.204, −0.043)	
General Health Questionnaire (GHQ-12) score (Ref: Non-distressed (0 ≤ GHQ-12 score ≤ 3))							0.471
Distressed (GHQ-12 score ≥ 4)	0.196	(−0.481, 0.872)	−0.120	(−0.763, 0.524)	0.356	(−0.174, 0.886)	
Number of prescribed medicines taken (Ref: 0 times)							0.015
1–2 medicines	−0.794	(−3.143, 1.555)	−0.335	(−0.864, 0.193)	−0.648	(−1.824, 0.528)	
≥ 3 medicines	−1.640	(−2.439, 0.841)	−0.860	(−1.900, 0.180)	−0.382	(−1.425, 0.661)	
Race/ethnicity (Ref: Non-White)							0.387
White 0.644		(−0.296, 1.583)	0.336	(−0.348, 1.021)	0.409	(−0.281, 1.100)	
Log Total household net income	−0.078	(−0.227, 0.071)	−0.079	(−0.199, 0.040)	−0.048	(−0.163, 0.068)	0.538
Year of last employment (Ref: Before 2008)							<0.001
2008	1.044	(0.162, 1.927)	0.996	(0.269, 1.722)	1.652	(0.788, 2.516)	
2009–2010	1.563	(0.772, 2.353)	1.587	(0.974, 2.200)	2.121	(1.312, 2.930)	
Number of children in housheold	0.148	(−0.222, 0.518)	0.017	(−0.171, 0.204)	0.150	(−0.085, 0.385)	0.544
Number of people in household	0.082	(−0.210, 0.373)	0.074	(−0.072, 0.221)	0.095	(−0.084, 0.274)	0.586

GCSE: General Certification of Secondary Education; CVD: Cardiovascular disease.

In summary, we found evidence that, compared with adults who remained unemployed, formerly unemployed adults who transitioned into poor quality jobs had elevated risks for a range of allostatic load biomarkers and the allostatic load index. In addition, we found little evidence of negative health selection into poor quality jobs. In contrast, physically healthier respondents without any disabilities at wave 1 were more likely to transition into good and poor quality jobs when compared with those who remained unemployed.

## Discussion

We found little evidence that re-employment into poor quality jobs was associated with better health and lower adverse levels of biomarkers related to chronic stress, compared with remaining unemployed. Instead, the evidence suggested that re-employment into poor quality jobs was associated with higher levels of chronic stress-related biomarkers compared with remaining unemployed. Furthermore, there was no evidence of negative health selection into poor quality work. Transitioning into good or poor quality jobs was associated with better physical health than remaining unemployed. Health-related selection is unlikely to explain why those who transition into poor quality jobs had higherer adverse levels of biomarkers related to chronic stress than those who remained unemployed.

The association between job quality and health and biomarkers related to stress has been found in a number of previous studies.^19^^,^^21^^,^^23^^,^^38^ What is new is the finding that poor job quality is associated with more adverse levels of biomarkers than remaining unemployed. This result is contrary to the belief that any job is good for health,^16^^,^^39^ and evidence that job loss during recessions is associated with increases in suicides.^40^ However, this apparent paradox may be partly explained by the differences in self-reported health measures and biomarkers. Those who transitioned into poor quality work had similar levels of mental health to those who remained unemployed, but the former had more adverse levels of biomarkers. Biomarkers are a measure of subclinical disease, potentially identifying those early in the pathological process towards overt clinical levels of disease and ill health.^41^ As most people are not aware of their biomarker levels unless these manifest in clinical symptoms or they regularly get health checks, there can be a disjunction between their self-perceptions of health and their subclinical biomarkers. If poor quality work results in more adverse levels of biomarkers, then those exposed to poor quality work may be on the pathway to manifesting metabolic- and cardiovascular-related diseases, at which point they may start reporting their health status as poor. These biological pathways are different from the pathways to suicide and violent deaths associated with unemployment during recessions.

As this is an observational study, we cannot make any causal claims. We have tried to show that health-related selection is unlikely to explain the pattern of results, but there may be other confounding factors related to unobserved heterogeneity in the job quality groups, which we have not taken into account. UKHLS did not measure any biomarkers at baseline (wave 1), so we were unable to look at changes in the biomarker levels between waves. The analytical sample sizes were quite small, especially for the biomarkers, although the recommended longitudinal survey weights were used in the analyses to compensate for missing biomarker data and other non-response biases. The allostatic load index in this study was constructed based on 12 available biomarkers from the UKHLS, and we lacked some key primary mediators of allostatic load.^20^ Despite these limitations, the study has a number of strengths. Job quality was measured using five dimensions of job quality,^24^ unlike most studies that only measure job satisfaction. Following up a cohort of formerly employed adults who were looking for work meant that the baseline samples were relatively homogeneous to start with. In addition, we controlled for baseline health and sociodemographic states in the analyses. The biomarkers used in the study, objective measures of health, were not affected by the method bias problem common in previous studies of job satisfaction and well-being.^11^^,^^16^^,^^17^^,^^19^

Despite the widespread belief that any employment, even poor quality work, is associated with better health and well-being than remaining unemployed, there is little evidence on whether becoming re-employed in poor quality work is better for health than remaining unemployed. The study finds some evidence that formerly unemployed adults who transitioned into poor quality work had higherer adverse levels of biomarkers compared with their peers who remained unemployed. The selection of the healthier unemployed adults into poor quality or stressful jobs was unlikely to explain their elevated levels of chronic stress-related biomarkers. Job quality cannot be disregarded in the employment success of the unemployed, and may have important implications for their health and well-being.

## Supplementary Data

Supplementary data are available at *IJE* online.

## Funding

This work was supported by the Economic and Social Research Council (ESRC ES/J019119/1, ES/M008592/1, ES/L008351/1).

**Conflict of interest:** None declared.

**Key Messages**

- Any job is not necessarily better than no job in relation to allostatic load biomarkers, because job quality is important.
- Job quality cannot be disregarded in the employment success of the unemployed.
- Just as ‘good work is good for health’, we must also remember poor quality work can be detrimental for health.

## Supplementary Material

Supplementary Data
