# Supplementary material for: Re-employment, job quality, health and allostatic load biomarkers: prospective evidence from the UK Household Longitudinal Study
Source: Int J Epidemiol. 2017 Aug 10;47(1):47–57. doi: 10.1093/ije/dyx150 (PMC5837779; doi:10.1093/ije/dyx150)
Supplement: Supplementary Data [file dyx150_ije-2016-12-1503-file005.docx]

**Supplementary 1a.** Full negative binomial regression models (allostatic load) and multiple regression models of health outcomes and related biomarkers at waves 2 and 3 regressed on job quality at wave 2 and covariates, among participants aged 30-75 years from Understanding Society, the UK: Coefficients (and 95% *CI*)

|  | **Allostatic load** |  | **Log HbA1c** |  | **Log Triglycerides** |  | **Log C-creative protein** |  |
| --- | --- | --- | --- | --- | --- | --- | --- | --- |
| **Variables** | *β (95%CI)* |  | *β (95%CI)* |  | *β (95%CI)* |  | *β (95%CI)* |  |
| Job adversity (Ref: remain unemployed) |  |  |  |  |  |  |  |  |
| Good quality job | -0.387(-1.004, 0.230 ) |  | 0.004(-0.109, 0.117) |  | -0.165(-0.35, 0.105) |  | -0.353(-0.852, 0.146) |  |
| One adverse measures | -0.262(-0.476, -0.047) |  | -0.020(-0.081, 0.042) |  | 0.029(-0.143, 0.201) |  | 0.183(-0.161, 0.527) |  |
| At least two adverse measures | 0.513(0.320, 0.706) |  | 0.057(-0.004, 0.117) |  | 0.389(0.220, 0.558) |  | 0.454(0.158, 0.749) |  |
| Age (Ref: 30-39 years) |  |  |  |  |  |  |  |  |
| 40-49 years | 0.440(0.164, 0.717) |  | -0.024(-0.151, 0.103) |  | 0.154(-0.087, 0.394) |  | 0.028(-0.278, 0.334) |  |
| 50-75 years | 0.696(0.452, 0.939) |  | 0.037(-0.132, 0.206) |  | 0.055(-0.219, 0.329) |  | 0.244(-0.054, 0.542) |  |
| Sex (Ref: Male) |  |  |  |  |  |  |  |  |
| Female | 0.007(-0.213, 0.227) |  | -0.062(-0.133, 0.008) |  | -0.135(-0.281, 0.012) |  | 0.245(-0.027, 0.518) |  |
| Highest qualification (Ref: Degree+Higher) |  |  |  |  |  |  |  |  |
| A level+GCSE | 0.174(-0.010, 0.359) |  | 0.091(0.006, 0.176) |  | 0.128(-0.044, 0.301) |  | 0.071(-0.173, 0.315) |  |
| Other+No qualification | 0.085(-0.114, 0.283) |  | 0.035(-0.027, 0.097) |  | 0.005(-0.169, 0.179) |  | 0.290(0.019, 0.561) |  |
| Housing tenure (Ref: Owned) |  |  |  |  |  |  |  |  |
| Council house | 0.080(-0.138, 0.297) |  | 0.013(-0.050, 0.076) |  | 0.099(-0.083, 0.280) |  | 0.077(-0.300, 0.455) |  |
| Rented | 0.099(-0.139, 0.337) |  | -0.041(-0.147, 0.064) |  | -0.148(-0.345, 0.050) |  | -0.016(-0.286, 0.255) |  |
| Marital status (Ref: Married) |  |  |  |  |  |  |  |  |
| Single | -0.131(-0.350, 0.088) |  | -0.025(-0.095, 0.045) |  | 0.037(-0.120, 0.193) |  | -0.019(-0.374, 0.335) |  |
| Separated, divorced or widowed | -0.029(-0.273, 0.214) |  | -0.023(-0.126, 0.080) |  | 0.126(-0.112, 0.364) |  | 0.198(-0.191, 0.588) |  |
| BMI | 0.045(0.026, 0.063) |  | 0.005(-0.001, 0.011) |  | 0.033(0.016, 0.049) |  | 0.046(0.022, 0.070) |  |
| Has CVD and/or diabetes (Ref: No) | 0.361(0.167, 0.556) |  | 0.081(-0.021, 0.184) |  | 0.270(0.087, 0.453) |  | 0.120(-0.212, 0.453) |  |
| SF-12 physical health composite scale scores | -0.007(-0.015, -0.0001) |  | -0.002(-0.007, 0.003) |  | 0.002(-0.009,0.012) |  | -0.013(-0.026, 0.0007) | |
| SF-12 mental health composite scale scores | -0.002(-0.010, 0.006) |  | -0.0005(-0.003, 0.002) |  | -0.005(-0.013, 0.002) |  | -0.004(-0.018, 0.009) | |
| Has long-term illness or impairment (Ref: No) | 0.032(-0.220, 0.284) |  | 0.005(-0.064, 0.074) |  | 0.015(-0.162, 0.191) |  | -0.003(-0.305, 0.300) |  |
| General Health Questionnaire (GHQ-12) score (Ref: Non-distressed) | -0.058(-0.297, 0.180) |  | -0.006(-0.071, 0.059) |  | 0.007(-0.160, 0.175) |  | -0.207(-0.484, 0.069) |  |
| Number of prescribed medicines taken (Ref: None) | |  |  |  |  |  |  |  |
| 1-2 medicines | 0.024(-0.265, 0.314) |  | 0.002(-0.062, 0.067) |  | -0.074(-0.266, 0.117) |  | 0.080(-0.291, 0.452) |  |
| ≥ 3 medicines | -0.034(-0.367, 0.299) |  | 0.046(-0.044, 0.136) |  | -0.173(-0.378, 0.032) |  | -0.059(-0.520, 0.402) |  |

**Supplementary 1a.** (Continued) Full negative binomial regression models (allostatic load) and multiple regression models of health outcomes and related biomarkers at waves 2 and 3 regressed on job quality at wave 2 and covariates, among participants aged 30-75 years from Understanding Society, the UK: Coefficients (and 95% CI)

|  | **Allostatic load** |  | **Log HbA1c** |  | **Log Triglycerides** |  | **Log C-creative protein** |
| --- | --- | --- | --- | --- | --- | --- | --- |
| **Variables** | *β (95%CI)* |  | *β (95%CI)* |  | *β (95%CI)* |  | *β (95%CI)* |
| Race/ethnicity (Ref: Non-White) |  |  |  |  |  |  |  |
| White | 0.183(-0.059, 0.425) |  | 0.055(-0.168, 0.278) |  | 0.100(-0.247, 0.448) |  | 0.029(-0.250, 0.309) |
| Log Total household net income | 0.008(-0.060, 0.076) |  | -0.005(-0.031, 0.021) |  | 0.067(-0.007, 0.142) |  | 0.023(-0.103, 0.149) |
| Year of last employment (Ref: Before 2008) |  |  |  |  |  |  |  |
| 2008 | 0.037(-0.178, 0.253) |  | -0.045(-0.130, 0.039) |  | -0.018(-0.209, 0.173) |  | -0.057(-0.328, 0.215) |
| 2009-2010 | -0.051(-0.275, 0.173) |  | -0.045(-0.118, 0.027) |  | 0.016(-0.117, 0.148) |  | -0.084(-0.406, 0.238) |
| Number of children in housheold | 0.119(-0.048, 0.286) |  | -0.028(-0.076, 0.020) |  | 0.039(-0.105, 0.183) |  | 0.023(-0.196, 0.241) |
| Number of people in household | -0.049(-0.166, 0.068) |  | 0.008(-0.042, 0.059) |  | -0.088(-0.203, 0.027) |  | 0.061(-0.103, 0.225) |
| Constant | -0.601(-1.605, 0.404) |  | 3.611(3.180, 4.041) |  | -0.694(-1.659, 0.271) |  | -0.636(-2.454, 1.182) |

**Supplementary 1b.** (Continued) Full linear regression models of health outcomes and related biomarkers at waves 2 and 3 regressed on job quality at wave 2 and covariates among participants aged 30-75 years from Understanding Society, the UK: Coefficients (and 95% *CI*)

|  | **Log Fibrinogen** |  | | **Log DHEA-S** |  | **Creatinine clearance rate** |  | **Total cholesterol-to-HDL ratio** | | | |
| --- | --- | --- | --- | --- | --- | --- | --- | --- | --- | --- | --- |
| **Variables** | *β (95%CI)* |  | | *β (95%CI)* |  | *β (95%CI)* |  | *β (95%CI)* | | | |
| Job adversity (Ref: remain unemployed) | |  | |  |  |  |  |  | | |  |
| Good quality job | -0.081(-0.191, 0.028) |  | | 0.182(-0.149, 0.513) |  | -2.634(-19.631, 14.363) |  | -0.151(-0.852, 0.551) | | |  |
| One adverse measures | -0.143(-0.237, -0.049) |  | | 0.012(-0.233, 0.257) |  | -1.645(-13.410, 10.120) |  | 0.025(-0.468, 0.518) | | |  |
| At least two adverse measures | 0.089(0.007, 0.170) |  | | -0.082(-0.305, 0.142) |  | -29.968(-35.910, -16.026) |  | 1.377(0.876, 1.878) | | |  |
| Age (Ref: 30-39 years) |  |  | |  |  |  |  |  | | |  |
| 40-49 years | 0.073(-0.028, 0.175) |  | -0.256(-0.559, 0.048) | |  | -19.788(-41.795, 2.219) |  | 0.217(-0.410, 0.848) | |  |  |
| 50-75 years | 0.113(-0.007, 0.232) |  | -0.598(-0.888, -0.309) | |  | -38.497(-65.438, -11.556) | -0.052(-0.723, 0.618) | |  |  |  |
| Sex (Ref: Male) |  |  |  | |  |  |  |  | |  |  |
| Female | 0.069(0.005, 0.134) |  | | -0.513(-0.708, -0.318) |  | -7.186(-16.951, 2.578) |  | -0.713(-1.132, -0.293) | |  |  |
| Highest qualification (Ref: Degree+Higher) | |  | |  |  |  |  |  | |  |  |
| A level+GCSE | -0.011(-0.093, 0.072) |  | | 0.094(-0.129, 0.317) |  | -2.550(-16.598, 11.499) |  | 0.074(-0.341, 0.489) | |  |  |
| Other+No qualification | 0.047(-0.024, 0.117) |  | | 0.122(-0.103, 0.347) |  | 1.352(-11.034, 13.740) |  | -0.248(-0.607, 0.110) | |  |  |
| Housing tenure (Ref: Owned) | |  | |  |  |  |  |  | |  |  |
| Council house | 0.119(0.015, 0.222) |  | | 0.086(-0.182, 0.355) |  | 2.535(-9.239, 14.310) |  | 0.543(0.081, 1.005) | | |  |
| Rented | 0.139(0.045, 0.234) |  | | 0.028(-0.232, 0.288) |  | -3.385(-21.376, 14.606) |  | 0.086(-0.522, 0.694) | | |  |
| Marital status (Ref: Married) | |  | |  |  |  |  |  | | |  |
| Single | 0.014(-0.068, 0.095) |  | | 0.069(-0.141, 0.280) |  | 3.550(-11.445, 18.545) |  | -0.029(-0.474, 0.415) | | |  |
| Separated, divorced or widowed | -0.046(-0.149, 0.056) |  | | 0.112(-0.138, 0.362) |  | 7.734(-10.069, 25.537) |  | 0.144(-0.416, 0.704) | | |  |
| BMI | 0.007(0.002, 0.013) |  | | -0.0003(-0.024, 0.023) |  | 3.154(1.668, 4.641) |  | 0.095(0.056, 0.134) | | |  |
| Has CVD and/or diabetes (Ref: No) | -0.005(-0.099, 0.088) |  | | 0.269(0.017, 0.521) |  | 3.741(-13.552, 21.034) |  | 0.369(-0.096, 0.834) | | |  |
| SF-12 physical health composite scale scores | 0.0006(-0.004, 0.005) |  | | 0.005(-0.014, 0.023) |  | -0.333(-1.006, 0.340) |  | 0.014(-0.006, 0.034) | | |  |
| SF-12 mental health composite scale scores | 0.004(0.001, 0.008) |  | | 0.0002(-0.007, 0.008) |  | -0.442(-0.923, 0.040) |  | -0.010(-0.023, 0.004) | | |  |
| Has long-term illness or impairment (Ref: No) | 0.039(-0.038, 0.117) |  | | -0.204(-0.413, 0.005) |  | 3.814(-9.191, 16.818) |  | -0.166(-0.640, 0.307) | | |  |
| General Health Questionnaire (GHQ-12) score (Ref: Non-distressed) | 0.042(-0.041, 0.126) |  | | -0.025(-0.285, 0.234) |  | 5.339(-7.893, 18.570) |  | 0.263(-0.155, 0.681) | | |  |
| Number of prescribed medicines taken (Ref; None) | | | |  |  |  |  |  | | |  |
| 1-2 medicines | 0.017(-0.063, 0.097) |  | | 0.119(-0.108, 0.346) |  | -10.429(-21.519, 0.661) |  | 0.235(-0.356, 0.827) | | |  |
| ≥ 3 medicines | 0.063(-0.044, 0.170) |  | | -0.245(-0.552, 0.022) |  | -14.164(-30.976, 2.647) |  | -0.349(-1.039, 0.340) | | |  |

**Supplementary 1b.** (Continued) Full linear regression models of health outcomes and related biomarkers at waves 2 and 3 regressed on job quality at wave 2 and covariates among participants aged 30-75 years from Understanding Society, the UK: Coefficients (and 95% *CI*)

|  | **Log Fibrinogen** |  | **Log DHEA-S** |  | **Creatinine clearance rate** |  | **Total cholesterol-to-HDL ratio** |
| --- | --- | --- | --- | --- | --- | --- | --- |
| **Variables** | *β (95%CI)* |  | *β (95%CI)* |  | *β (95%CI)* |  | *β (95%CI)* |
| Race/ethnicity (Ref: Non-White) |  |  |  |  |  |  |  |
| White | -0.027(-0.167, 0.112) |  | -0.172(0.508, 0.164) |  | 5.242(-22.978, 33.463) |  | -0.197(-1.063, 0.670) |
| Log Total household net income | -0.004(-0.034, 0.026) |  | -0.010(-0.115, 0.095) |  | 0.395(-4.919, 5.710) |  | -0.089(-0.291, 0.113) |
| Year of last employment (Ref: Before 2008) |  |  |  |  |  |  |  |
| 2008 | -0.021(-0.097, 0.056) |  | -0.070(-0.289, 0.149) |  | -1.664(-14.981, 11.652) |  | -0.171(-0.705, 0.363) |
| 2009-2010 | 0.064(-0.022, 0.150) |  | -0.065(-0.319, 0.189) |  | 10.386(-2.245, 23.017) |  | -0.499(-0.978, -0.020) |
| Number of children in housheold | -0.026(-0.093, 0.040) |  | -0.025(-0.200, 0.150) |  | -0.448(-10.537, 9.640) |  | -0.162(-0.555, 0.230) |
| Number of people in household | 0.012(-0.043, 0.068) |  | -0.013(-0.156, 0.129) |  | -1.009(-9.987, 7.970) |  | 0.170(-0.131, 0.471) |
| Constant | 0.374(-0.226, 0.974) |  | 1.786(0.145, 3.428) |  | 93.879(18.359, 169.399) |  | 1.679(-0.324, 3.681) |

**Supplementary 1c.** (Continued) Full linear regression models of health outcomes and related biomarkers at waves 2 and 3 regressed on job quality at wave 2 and covariates among participants aged 30-75 years from Understanding Society, the UK: Coefficients (and 95% *CI*)

|  | **Log Insulin-like growth factor 1** |
| --- | --- |
| **Variables** | *β (95%CI)* |
| Job adversity (Ref: remain unemployed) |  |
| Good quality job | 0.025(-0.205, 0.256) |
| One adverse measures | 0.067(-0.041, 0.174) |
| At least two adverse measures | 0.016(-0.088, 0.120) |
| Age (Ref: 30-39 years) |  |
| 40-49 years | -0.004(-0.146, 0.139) |
| 50-75 years | -0.194(-0.323, -0.066) |
| Sex (Ref: Male) |  |
| Female | -0.033(-0.131, 0.064) |
| Highest qualification (Ref: Degree+Higher) |  |
| A level+GCSE | -0.068(-0.182, 0.046) |
| Other+No qualification | -0.016(-0.146, 0.114) |
| Housing tenure (Ref: Owned) |  |
| Council house | -0.028(-0.152, 0.095) |
| Rented | -0.037(-0.198, 0.123) |
| Marital status (Ref: Married) |  |
| Single | 0.149(0.006, 0.292) |
| Separated, divorced or widowed | 0.039(-0.130, 0.208) |
| BMI | -0.003(-0.017, 0.011) |
| Has CVD and/or diabetes (Ref: No) | 0.022(-0.086, 0.129) |
| SF-12 physical health composite scale scores | 0.006(0.0004, 0.011) |
| SF-12 mental health composite scale scores | 0.003(-0.002, 0.008) |
| Has long-term illness or impairment (Ref: No) | 0.115(-0.020, 0.249) |
| General Health Questionnaire (GHQ-12) score (Ref: Non-distressed) | -0.011(-0.141, 0.120) |
| Number of prescribed medicines taken (Ref; None) | |
| 1-2 medicines | 0.041(-0.091, 0.172) |
| ≥ 3 medicines | 0.055(-0.131, 0.241) |
| Race/ethnicity (Ref: Non-White) |  |
| White | -0.114(-0.256, 0.029) |
| Log Total household net income | 0.002(-0.055, 0.059) |
| Year of last employment (Ref: Before 2008) |  |
| 2008 | -0.022(-0.180, 0.136) |
| 2009-2010 | -0.014(-0.120, 0.092) |
| Number of children in housheold | -0.044(-0.115, 0.027) |
| Number of people in household | 0.018(-0.044, 0.080) |
| Constant | 2.424(1.640, 3.208) |

GCSE: General Certification of Secondary Education; CVD: Cardiovascular disease

Fully-adjusted models were fitted by adjusting for age, gender, highest qualification, housing tenure, marital status, BMI, has CVD and/or diabetes or not, SF-12 physical health composite scale scores, SF-12 mental health composite scale scores, Has long-term illness or impairment, General Health Questionnaire (GHQ-12) score , number of prescribed medicines taken, log transformation of household net income, race/ethnicity, number of children within household, number of people within household and year of last employment.

**Supplementary 2.** Full multiple regression models of blood pressure and anthropometry measures at waves 2 and 3 regressed on job quality at wave 2 and covariates among participants aged 30-75 years from Understanding Society, the UK: Coefficients (and 95% *CI*)

|  | **Log Systolic blood pressure** |  | **Log Diastolic blood pressure** |  | **Waist-to-height ratio** |  | **Log Omron valid mean pulse** |
| --- | --- | --- | --- | --- | --- | --- | --- |
| **Variables** | *β (95%CI)* |  | *β (95%CI)* |  | *β (95%CI)* |  |  |
| Job adversity (Ref: remain unemployed) |  |  |  |  |  |  |  |
| Good quality job | -0.023(-0.068, 0.023) |  | -0.029(-0.086, 0.027) |  | -0.020(-0.041, 0.0006) |  | -0.010(-0.120, 0.099) |
| One adverse measures | -0.019(-0.052, 0.014) |  | -0.010(-0.056, 0.037) |  | -0.007(-0.022, 0.008) |  | -0.010(-0.075, 0.056) |
| At least two adverse measures | 0.028(-0.018, 0.074) |  | 0.010(-0.071, 0.091) |  | -0.003(-0.022, 0.017) |  | -0.029(-0.107, 0.049) |
| Age (Ref: 30-39 years) |  |  |  |  |  |  |  |
| 40-49 years | 0.048(0.009, 0.087) |  | 0.075(0.008, 0.143) |  | -0.001(-0.018, 0.016) |  | 0.040(-0.039, 0.118) |
| 50-75 years | 0.116(0.079, 0.153) |  | 0.092(0.036, 0.148) |  | 0.018(0.001, 0.035) |  | 0.002(-0.076, 0.080) |
| Sex (Ref: Male) |  |  |  |  |  |  |  |
| Female | -0.037(-0.066, -0.008) |  | -0.0008(-0.039, 0.037) |  | -0.008(-0.018, 0.001) |  | 0.033(-0.009, 0.076) |
| Highest qualification (Ref: Degree+Higher) |  |  |  |  |  |  |  |
| A level+GCSE | 0.002(-0.032, 0.036) |  | 0.004(-0.035, 0.043) |  | 0.005(-0.008, 0.017) |  | 0.019(-0.042, 0.080) |
| Other+No qualification | 0.023(-0.017, 0.063) |  | 0.021(-0.035, 0.076) |  | 0.011(-0.003, 0.025) |  | -0.051(-0.107, 0.004) |
| Housing tenure (Ref: Owned) |  |  |  |  |  |  |  |
| Council house | -0.001(-0.042, 0.040) |  | 0.024(-0.027, 0.074) |  | 0.0005(-0.018, 0.019) |  | 0.053(-0.013, 0.120) |
| Rented | 0.019(-0.041, 0.079) |  | 0.004(-0.059, 0.067) |  | 0.0002(-0.017, 0.017) |  | 0.058(-0.036, 0.152) |
| Marital status (Ref: Married) |  |  |  |  |  |  |  |
| Single | 0.007(-0.033, 0.047) |  | -0.0002(-0.052, 0.052) |  | -0.005(-0.018, 0.008) |  | -0.038(-0.113, 0.037) |
| Separated, divorced or widowed | -0.013(-0.053, 0.027) |  | -0.029(-0.085, 0.028) |  | 0.0005(-0.020, 0.021) |  | -0.010(-0.091, 0.071) |
| BMI | 0.004(-0.0005, 0.008) |  | 0.006(0.002, 0.011) |  | 0.013(0.011, 0.014) |  | -0.003(-0.008, 0.002) |
| Has CVD and/or diabetes (Ref: No) | 0.070(0.030, 0.109) |  | 0.039(-0.008, 0.085) |  | 0.018(0.007, 0.029) |  | 0.002(-0.064, 0.068) |
| SF-12 physical health composite scale scores | 0.001(-0.0004, 0.003) |  | 0.001(-0.001, 0.004) |  | -0.0003(-0.0009, 0.0003) |  | -0.002(-0.005, 0.001) |
| SF-12 mental health composite scale scores | 0.0003(-0.0009, 0.002) |  | -0.0002(-0.002, 0.001) |  | -0.000006(-0.0006, 0.0006) |  | -0.001(-0.003, 0.0006) |
| Has long-term illness or impairment (Ref: No) | 0.014(-0.040, 0.068) |  | 0.021(-0.033, 0.076) |  | -0.011(-0.024, 0.002) |  | 0.012(-0.049, 0.073) |
| GHQ-12 score (Ref: Non-distressed) | 0.007(-0.022, 0.036) |  | 0.006(-0.043, 0.054) |  | 0.009(-0.008, 0.025) |  | -0.022(-0.080, 0.037) |
| Number of prescribed medicines taken (Ref: None) |  |  |  |  |  |  |  |
| 1-2 medicines | -0.036(-0.075, 0.003) |  | -0.035(-0.081, 0.011) |  | -0.009(-0.023, 0.006) |  | -0.023(-0.084, 0.037) |
| ≥ 3 medicines | -0.053(-0.111, 0.005) |  | -0.069(-0.134, -0.005) |  | 0.010(-0.005, 0.026) |  | -0.020(-0.088, 0.048) |

**Supplementary 2.** (Continued) Full linear regression models of blood pressure and anthropometry measures at waves 2 and 3 regressed on job quality at wave 2 and covariates among participants aged 30-75 years from Understanding Society, the UK: Coefficients (and 95% *CI*)

|  | **Log Systolic blood pressure** |  | **Log Diastolic blood pressure** |  | **Waist-to-height ratio** |  | **Log Omron valid mean pulse** |
| --- | --- | --- | --- | --- | --- | --- | --- |
| **Variables** | *β (95%CI)* |  | *β (95%CI)* |  | *β (95%CI)* |  |  |
| Race/ethnicity (Ref: Non-White) |  |  |  |  |  |  |  |
| White | -0.022(-0.074, 0.030) |  | -0.007(-0.068, 0.055) |  | 0.003(-0.013, 0.018) |  | 0.004(-0.088, 0.096) |
| Log Total household net income | -0.007(-0.014, 0.0004) |  | -0.003(-0.012, 0.005) |  | 0.007(-0.004, 0.010) |  | -0.005(-0.014, 0.005) |
| Year of last employment (Ref: Before 2008) |  |  |  |  |  |  |  |
| 2008 | 0.019(-0.017, 0.055) |  | 0.002(-0.063, 0.068) |  | 0.004(-0.010, 0.019) |  | 0.027(-0.043, 0.096) |
| 2009-2010 | 0.037(-0.010, 0.084) |  | 0.079(0.027, 0.130) |  | 0.004(-0.009, 0.017) |  | 0.018(-0.049, 0.084) |
| Number of children in housheold | 0.006(-0.024, 0.037) |  | 0.020(-0.017, 0.058) |  | 0.004(-0.009, 0.017) |  | 0.016(-0.035, 0.066) |
| Number of people in household | 0.0006(-0.020, 0.022) |  | 0.002(-0.026, 0.029) |  | -0.004(-0.013, 0.005) |  | -0.005(-0.042, 0.032) |
| Constant | 4.608(4.408, 4.808) |  | 4.005(3.718, 4.292) |  | 0.184(0.113, 0.256) |  | 4.503(4.161, 4.844) |

GCSE: General Certification of Secondary Education; CVD: Cardiovascular disease; GHQ: General Health Questionnaire

Fully-adjusted models were fitted by adjusting for age, gender, highest qualification, housing tenure, marital status, BMI, has CVD and/or diabetes or not, SF-12 physical health composite scale scores, SF-12 mental health composite scale scores, Has long-term illness or impairment, General Health Questionnaire (GHQ-12) score , number of prescribed medicines taken, log transformation of household net income, race/ethnicity, number of children within household, number of people within household and year of last employment.

**Supplementary 3.** Full linear regression models of self-reported health measures at waves 2 and 3 regressed on job quality at wave 2 and covariates among participants aged 30-75 years from Understanding Society, the UK: Coefficients (and 95% *CI*)

|  | **SF-12 physical component score** |  | **SF-12 mental component score** |  | **Log Total household net income** |  |
| --- | --- | --- | --- | --- | --- | --- |
| **Variables** | *β (95%CI)* |  | *β (95%CI)* |  | *β (95%CI)* |  |
| Job adversity (Ref: remain unemployed) |  |  |  |  |  |  |
| Good quality job | -0.701(-4.611, 3.209) |  | 5.541(-2.841, 13.923) |  | 0.402(-0.146, 0.949) |  |
| One adverse measures | -0.490(-1.671, 0.691) |  | 3.103(0.966, 5.240) |  | 0.320(-0.008, 0.648) |  |
| At least two adverse measures | 1.914(-3.599, 7.426) |  | 2.299(-2.406, 7.005) |  | 0.408(0.037, 0.779) |  |
| Age (Ref: 30-39 years) |  |  |  |  |  |  |
| 40-49 years | 1.025(-2.751, 4.801) |  | 1.014(-3.871, 5.899) |  | 0.129(-0.171, 0.429) |  |
| 50-75 years | -0.813(-3.845, 2.218) |  | 1.973(-3.304, 7.251) |  | 0.035(-0.213, 0.284) |  |
| Sex (Ref: Male) |  |  |  |  |  |  |
| Female | 0.584(-2.186, 3.354) |  | 0.282(-2.834, 3.398) |  | -0.002(-0.306, 0.301) |  |
| Highest qualification (Ref: Degree+Higher) |  |  |  |  |  |  |
| A level+GCSE | -0.033(-2.392, 2.236) |  | -1.472(-3.880, 0.935) |  | 0.040(-0.321, 0.402) |  |
| Other+No qualification | -2.239(-4.280, -0.198) |  | -0.827(-5.159, 3.505) |  | -0.002(-0.241, 0.238) |  |
| Housing tenure (Ref: Owned) |  |  |  |  |  |  |
| Council house | -0.607(-4.547, 3.333) |  | -1.473(-5.691, 2.745) |  | -0.193(-0.593, 0.207) |  |
| Rented | 0.934(-1.470, 3.338) |  | -1.837(-5.783, 2.110) |  | -0.179(-0.517, 0.160) |  |
| Marital status (Ref: Married) |  |  |  |  |  |  |
| Single | -0.755(-3.456, 1.945) |  | 2.524(-0.605, 5.653) |  | -0.091(-0.245, 0.063) |  |
| Separated, divorced or widowed | -1.632(-3.760, 0.496) |  | -1.689(-4.966, 1.588) |  | -0.341(-0.991, 0.308) |  |
| BMI | -0.068(-0.381, 0.245) |  | -0.013(-0.451, 0.425) |  | 0.003(-0.012, 0.019) |  |
| Has CVD and/or diabetes (Ref: No) | 0.383(-1.652, 2.419) |  | 2.246(-0.842, 5.333) |  | -0.117(-0.280, 0.047) |  |
| SF-12 physical health composite scale scores | 0.712(0.607, 0.816) |  | 0.014(-0.122, 0.150) |  | 0.001(-0.008, 0.010) |  |
| SF-12 mental health composite scale scores | 0.157(0.046, 0.268) |  | 0.445(0.243, 0.647) |  | 0.001(-0.018, 0.021) |  |
| Has long-term illness or impairment (Ref: No) | -1.645(-3.765, 0.476) |  | 0.208(-3.181, 3.598) |  | -0.032(-0.242, 0.179) |  |
| General Health Questionnaire (GHQ-12) score (Ref: Non-distressed) | 0.096(-3.167, 3.360) |  | -4.504(-8.424, -0.584) |  | -0.103(-0.306, 0.100) |  |
| Number of prescribed medicines taken (Ref: None) | |  |  |  |  |  |
| 1-2 medicines | 0.068(-2.800, 2.935) |  | -1.193(-5.437, 3.051) |  | -0.080(-0.406, 0.246) |  |
| ≥ 3 medicines | -1.000(-4.012, 2.012) |  | -2.094(-8.264, 4.076) |  | 0.246(-0.164, 0.656) |  |

**Supplementary 3.** (Continued) Full linear regression models of self-reported health measures at waves 2 and 3 regressed on job quality at wave 2 and covariates among participants aged 30-75 years from Understanding Society, the UK: Coefficients (and 95% *CI*)

|  | **SF-12 physical component score** |  | **SF-12 mental component score** |  | **Log Total household net income** |
| --- | --- | --- | --- | --- | --- |
| **Variables** | *β (95%CI)* |  | *β (95%CI)* |  | *β (95%CI)* |
| Race/ethnicity (Ref: Non-White) |  |  |  |  |  |
| White | -4.325(-8.154, -0.497) |  | 0.676(-4.628, 5.981) |  | 0.045(-0.303, 0.393) |
| Log Total household net income | 0.701(0.085, 1.316) |  | -0.361(-1.432, 0.710) |  | 0.503(0.443, 0.563) |
| Year of last employment (Ref: Before 2008) |  |  |  |  |  |
| 2008 | -0.242(-2.715, 2.231) |  | 0.663(-3.062, 4.388) |  | -0.122(-0.302, 0.058) |
| 2009-2010 | -1.869(-5.096, 1.359) |  | 0.415(-4.244, 5.073) |  | -0.106(-0.395, 0.183) |
| Number of children in housheold | 1.280(-1.094, 3.653) |  | 0.230(-3.228, 3.687) |  | -0.017(-0.162, 0.128) |
| Number of people in household | -1.349(-2.792, 0.095) |  | -0.605(-3.590, 2.380) |  | 0.042(-0.103, 0.187) |
| Constant | 7.828(-6.877, 22.532) |  | 30.441(13.283, 47.600) |  | 3.528(1.621, 5.435) |

GCSE: General Certification of Secondary Education; CVD: Cardiovascular disease

Fully-adjusted models were fitted by adjusting for age, gender, highest qualification, housing tenure, marital status, BMI, has CVD and/or diabetes or not, SF-12 physical health composite scale scores, SF-12 mental health composite scale scores, Has long-term illness or impairment, General Health Questionnaire (GHQ-12) score , number of prescribed medicines taken, log transformation of household net income, race/ethnicity, number of children within household, number of people within household and year of last employment.

**Supplementary 4.** Negative binomial regression models of allostatic load at waves 2 and 3 regressed on individual job quality measures at wave 2 and covariates among participants aged 30-75 years from Understanding Society, the UK: Coefficients (and 95% *CI*)

|  | **Allostatic load** |  | *Overall P-value* |
| --- | --- | --- | --- |
| **Variables** | *β (95%CI)* |  |  |
| Job satisfaction ^a^ (Ref: Remain unemployed) |  |  | 0.007 |
| Not satisfactory | 0.359(0.116, 0.603) |  |  |
| Satisfactory | -0.073(-0.285, 0.139) |  |  |
| Job anxiety ^b^ (Ref: Remain unemployed) |  |  | 0.00003 |
| Anxious | 0.373(0.175, 0.571) |  |  |
| Not anxious | -0.148(-0.376, 0.080) |  |  |
| Job automony ^c^ (Ref: Remain unemployed) |  |  | 0.089 |
| Low autonomy | 0.171(0.0003, 0.341) |  |  |
| High autonomy | -0.109(-0.413, 0.194) |  |  |
| Job insecurity ^d^ (Ref: Remain unemployed) |  |  | 0.922 |
| Insecure | -0.016(-0.411, 0.380) |  |  |
| Secure | 0.046(-0.195, 0.286) |  |  |
| Low job pay ^e^ (Ref: Remain unemployed) |  |  | 0.0004 |
| Low Pay | 0.549(0.236, 0.863) |  |  |
| High pay | -0.208(-0.466, 0.051) |  |  |

^a^ Job satisfaction was measured on a 7-point Likert-scale (where 1=completely dissatisfied and 7=completely satisfied). We used 4 as the cut-off to define low job satisfaction.

^b^ Job anxiety was derived as the mean of six questions on job-related wellbeing, e.g., how much of the time in the past week one feels tense/uneasy/worried/depressed/gloomy/miserable about job. Each question was scored 1 (never), 2 (occasionally), 3 (some of the time), 4 (most of the time) and 5 (all of the time). A cut-off of 2 or higher reflects some job anxiety.

^c^ Job autonomy was computed as the mean of five questions on how much influence a participant has over tasks, workplace, work manner, task order and work hours in his/her current job. Each question was measured on a four-point scale from 1 (a lot), 2 (some), 3 (a little) and 4 (none). A cut-off of 2 or higher represents a low autonomy job.

^d^ Job insecurity was measured by a question on 'how likely you think it is that you will lose your job during the next 12 months'. The four response categories were grouped into two: low job security (very likely or likely) and high job security (unlikely or very unlikely).

^e^ Low job pay was defined as being in the lowest quartile of hourly pay which was calculated from usual gross pay per month and number of hours per week.

Fully-adjusted models were fitted by adjusting for age, gender, highest qualification, housing tenure, marital status, BMI, has CVD and/or diabetes or not, SF-12 physical health composite scale scores, SF-12 mental health composite scale scores, Has long-term illness or impairment, General Health Questionnaire (GHQ-12) score , number of prescribed medicines taken, log transformation of household net income, race/ethnicity, number of children within household, number of people within household and year of last employment.
